# Waldenström macroglobulinemia presenting with lumbar vertebral fracture as the initial symptom: a rare case report and literature review

**DOI:** 10.3389/fmed.2026.1947916

**Published:** 2026-10-23

**Authors:** Li Xu, Chen Shen, Jian Mi, Zihan Hu, Qifeng Sheng, Pengfei Zhu, Zhuorun Song, Feng Zhou

**Affiliations:** Department of Orthopedics, The First Affiliated Hospital of Soochow University, Suzhou, China

**Keywords:** low back pain, lumbar fracture, osteolytic lesion, pathological fracture, Waldenström macroglobulinemia

## Abstract

Waldenström macroglobulinemia (WM) is a rare type of B-cell lymphoma characterized by lymphoplasmacytic infiltration in the bone marrow and elevated serum monoclonal immunoglobulin M (IgM). Pathological bone-destructive lesions are exceedingly uncommon in this disease. Cases of WM presenting initially with lumbar vertebral fractures have been sporadically reported in clinical practice. We also outline the features that distinguish WM-related fractures from those caused by multiple myeloma and skeletal metastases, and summarize 12 previously published cases identified by a systematic PubMed search.

## 1 Introduction

Waldenström Macroglobulinemia (WM) is a rare B-cell lymphoma characterized by lymphoplasmacytic infiltration in the bone marrow and elevated serum monoclonal immunoglobulin M (IgM) (1), accounting for approximately 1%–2% of all hematologic malignancies, with an annual incidence of about three per million individuals. Clinically overt osteolytic lesions and pathological fractures are uncommon in WM, and their presence has traditionally been regarded as a feature favouring multiple myeloma (2, 3). The reported frequency of skeletal involvement, however, depends strongly on how patients are imaged. Cases in which a vertebral fracture is the presenting event leading to the diagnosis of WM remain rare.

## 2 Case presentation

In January 2026, a 77-year-old male patient was admitted to our hospital with a one-year history of recurrent low back pain of unknown cause, which had aggravated with limited mobility over the preceding month. Physical examination revealed localized tenderness and percussion pain around the L2 spinous process. Computed Tomography (CT) revealed disruption of cortical bone continuity in the L1 and L2 vertebrae, although the patient's bone mineral density did not meet the criteria for osteoporosis (Figure 1).

**Figure 1:**
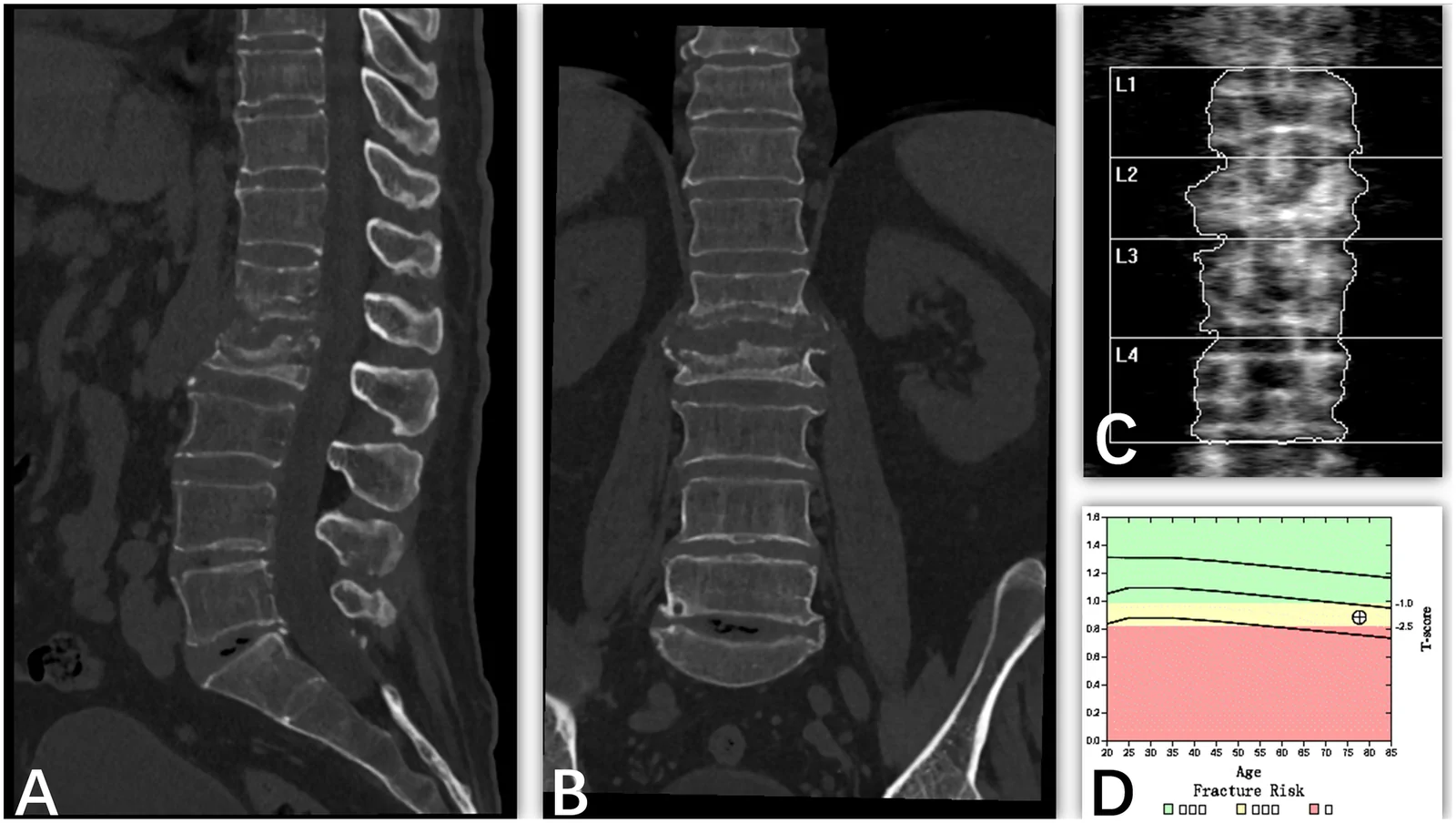
Computed tomography in the coronal **(A)** and sagittal **(B)** planes reveals osteolytic changes in the patient's L1 and L2 vertebrae, with significant loss of vertebral body height. The patient's bone mineral density is at the level of osteopenia **(C,D)**.

As part of our institutional work-up for suspected pathological vertebral fracture in elderly patients, and given that skeletal metastasis from solid tumours is the most frequent cause of osteolytic vertebral fracture in this age group, serum tumour markers were obtained as a rapid, non-invasive triage test in parallel with serum protein electrophoresis (SPEP) and immunofixation electrophoresis (IFE), rather than as a stand-alone diagnostic test. Complete blood count, serum calcium, creatinine, albumin, lactate dehydrogenase and *β*2-microglobulin were obtained at the same time. Tumour markers including CEA, AFP and PSA were within reference ranges, making a metastatic carcinoma less likely, while definitive diagnosis relied on histopathology. SPEP showed a prominent M-spike in the *γ* region (Figure 2A). On IFE, the IgM lane showed a single narrow band at the position of the M-spike, the *κ* lane showed intense staining at the same position, the IgG lane showed only broad diffuse (polyclonal) staining, and the IgA lane showed faint diffuse staining without a band (Figure 2B). Quantitative analysis showed markedly elevated serum IgM (23.10 g/L; reference 0.30–2.20 g/L), normal IgG (11.00 g/L; reference 8.60–17.40 g/L) and decreased IgA (0.74 g/L; reference 1.00–4.20 g/L). Total *κ* light chain was elevated (23.30 g/L), whereas total *λ* light chain was normal (4.42 g/L). Serum free *κ* light chain was 416.94 mg/L, with a free *κ*/*λ* ratio of 11.659 (reference 0.260–1.650), indicating a monoclonal IgM-*κ* paraprotein (Table 1). The M-spike was not quantified densitometrically; the nephelometric serum IgM concentration was therefore used as the measure of paraprotein burden. A monoclonal IgM paraprotein, rather than an IgG or IgA paraprotein, is atypical for the pathological fractures associated with multiple myeloma.

**Figure 2:**
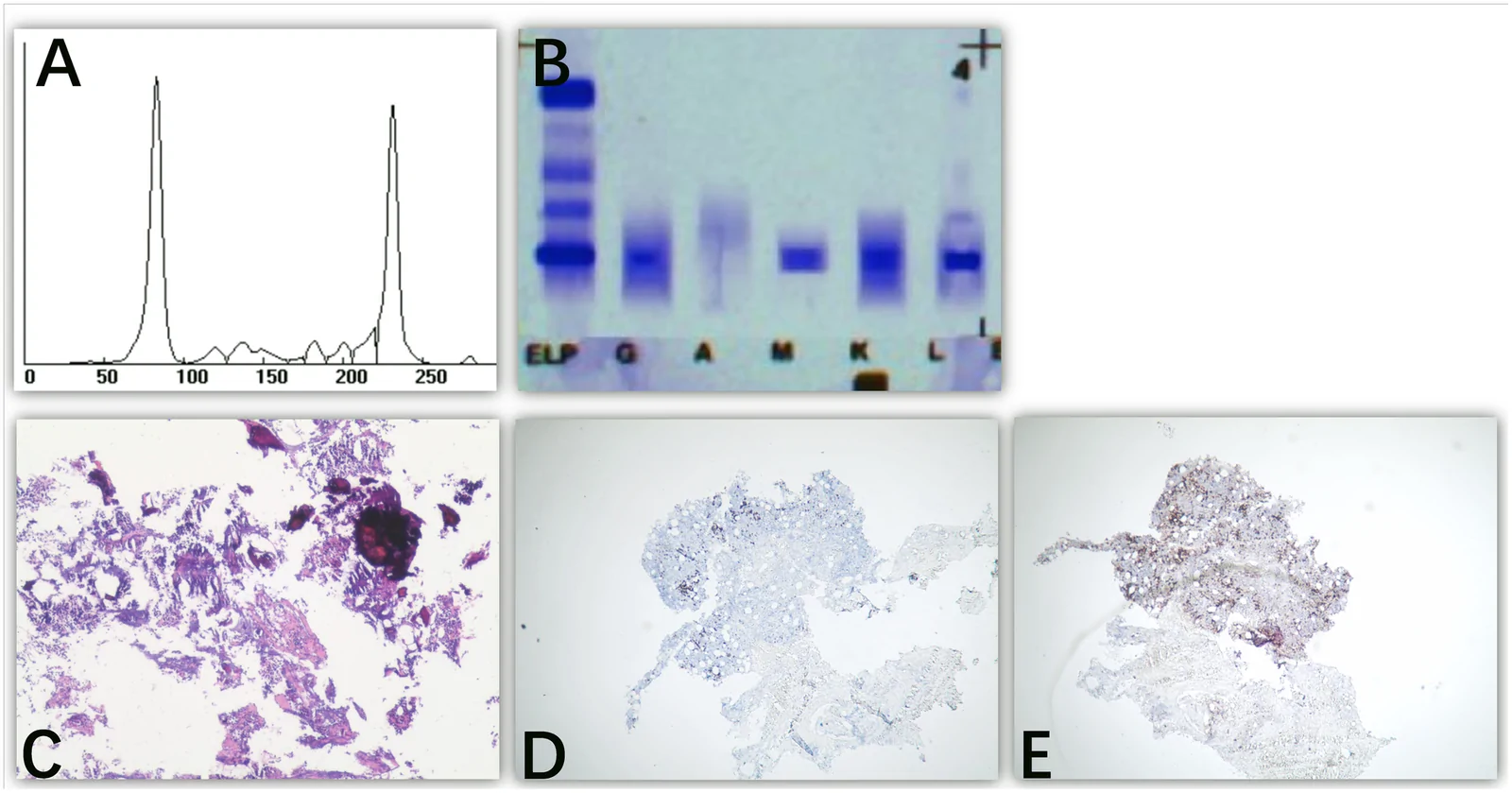
**(A)** serum protein electrophoresis showing a prominent M-spike in the *γ* region. **(B)** Serum immunofixation electrophoresis showing a narrow band in the IgM lane at the position of the M-spike and intense staining in the *κ* lane at the same position, with broad, diffuse (polyclonal) staining in the IgG lane and faint diffuse staining in the IgA lane; Histopathological examination of vertebral puncture specimens revealed bone sequestra and myeloid cells (C, HE staining), with plasma cells accounting for 6%. Immunohistochemical staining demonstrated partial positivity for CD38 **(D)** and CD138 **(E).**

**Table 1:** Serum immunoglobulin and light-chain quantification at diagnosis.

Item	Result	Flag	Reference range
Immunoglobulin G (IgG)	11.00 g/L	-	8.60–17.40 g/L
Immunoglobulin A (IgA)	0.74 g/L	↓	1.00–4.20 g/L
Immunoglobulin M (IgM)	23.10 g/L	↑	0.30–2.20 g/L
Total *κ* light chain	23.30 g/L	↑	6.29–13.50 g/L
Total *λ* light chain	4.42 g/L	-	3.13–7.23 g/L
Free κ light chain	416.94 mg/L	↑	3.30–19.40 mg/L
Free λ light chain	35.76 mg/L	↑	5.71–26.30 mg/L
Free κ/λ ratio	11.659	↑	0.260–1.650

For further diagnosis, the patient was hospitalized and underwent plain and contrast-enhanced lumbar spine Magnetic Resonance Imaging (MRI). The contrast-enhanced MRI revealed annular enhancement at the fracture margins, which presents a slight distinction from the typical diffuse vertebral infiltration observed in most pathological fractures associated with multiple myeloma (Figure 3).

**Figure 3:**
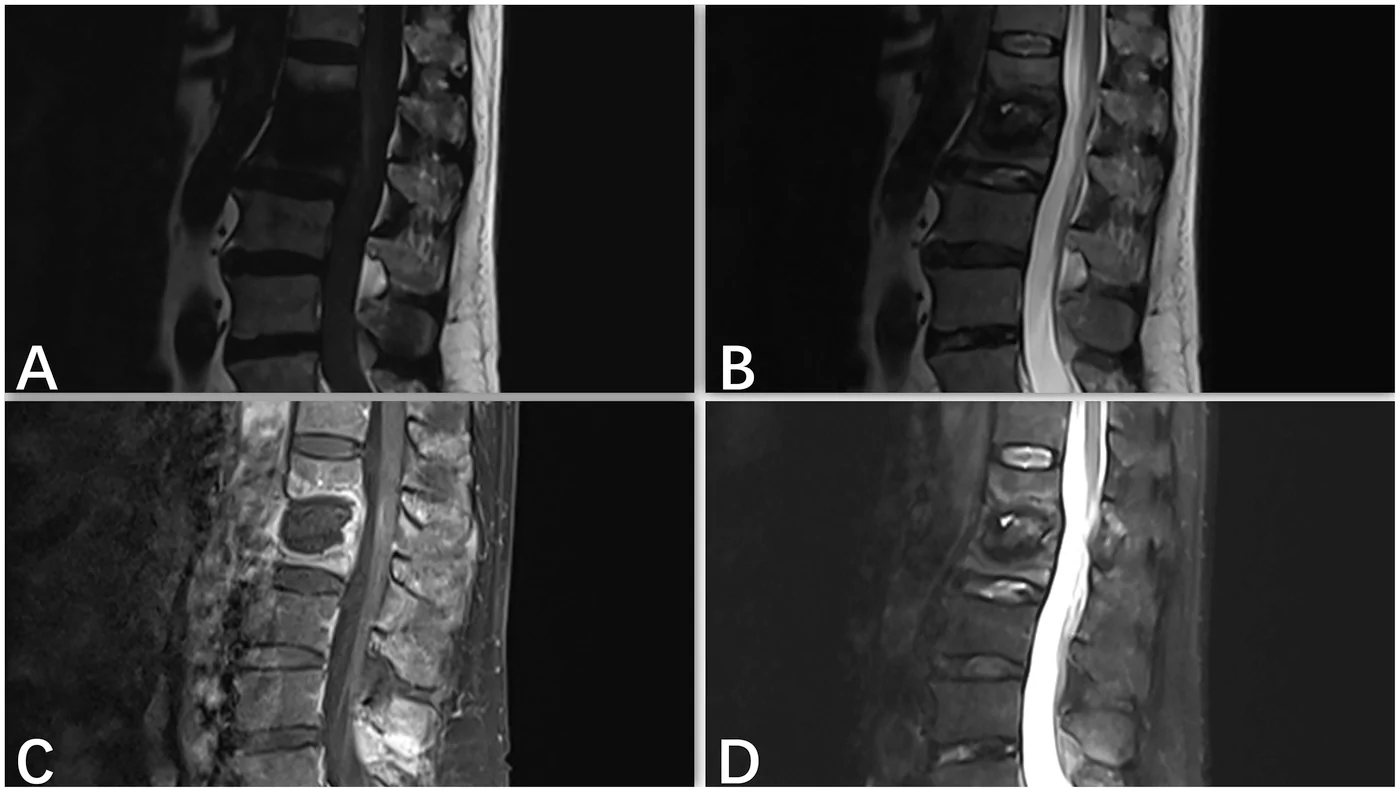
**(A)** sagittal T1-weighted MRI; **(B)** T2-weighted MRI, showing fusion of the vertebral body margins; **(C)** enhanced T1-weighted MRI, which reveals annular enhancement at the fracture margins; **(D)** T2-weighted fat-suppressed MRI.

The patient subsequently underwent internal fixation surgery for the lumbar vertebral fracture, during which a partial tissue biopsy was harvested from the fracture site under C-arm fluoroscopy guidance for pathological examination. The procedure achieved rigid internal fixation between the vertebrae adjacent to the fractured segment, with no intraoperative complications observed. The surgical specimens were submitted for pathological analysis.

Postoperative histopathological examination of the vertebral specimen revealed bone sequestra and marrow tissue containing lymphoid cells and plasma cells; plasma cells accounted for approximately 6% of the cellularity without sheets of atypical or immature plasma cells. Immunohistochemistry showed CD38 (partially +), CD138 (focally +), MUM1 (focally +), *κ* light chain (partially +), *λ* light chain (focally +) and CK (−) (Figures 2C–E). No light-chain restriction was demonstrated on this decalcified specimen.

After undergoing orthopedic surgery, the patient was referred to the hematology department for further diagnosis and treatment. Subsequently, a bone marrow puncture and biopsy were performed. The bone marrow aspirate (200 nucleated cells counted) was hypercellular, with a granulocyte-to-erythroid ratio of 0.77:1 and more than 300 megakaryocytes per smear. Lymphocytes accounted for 24.0% of nucleated cells (reference 7.59%–33.46%); most had irregular cell outlines with pseudopod-like cytoplasmic projections, and clusters of lymphocytes were present. Plasma cells were easily found (1.0%), including binucleated forms, and no blasts were seen. The morphological findings were considered suggestive of WM. A trephine histology report describing the pattern and extent of infiltration was not available, and MYD88 L265P mutation testing was not performed. On the basis of the monoclonal IgM-*κ* paraprotein, the clustered, morphologically atypical marrow lymphocytes accompanied by plasma cells, the CD5(−)CD10(−) clonal B-cell population and the absence of clonal plasma cells, the diagnosis was considered most consistent with WM, and IgM myeloma was excluded. Subsequently, the patient was started on targeted therapy with zanubrutinib, a next-generation Bruton's tyrosine kinase (BTK) inhibitor.

Postoperative CT confirmed stable instrumentation placement in the patient's lumbar spine. Follow-ups at one and three months revealed no evidence of new fractures or tumor infiltration (Figure 4). Currently, the patient remains in stable condition. The final diagnosis was WM (IgM-*κ*) with an osteolytic pathological fracture of L1–L2 attributed to probable osseous involvement by WM.

**Figure 4:**
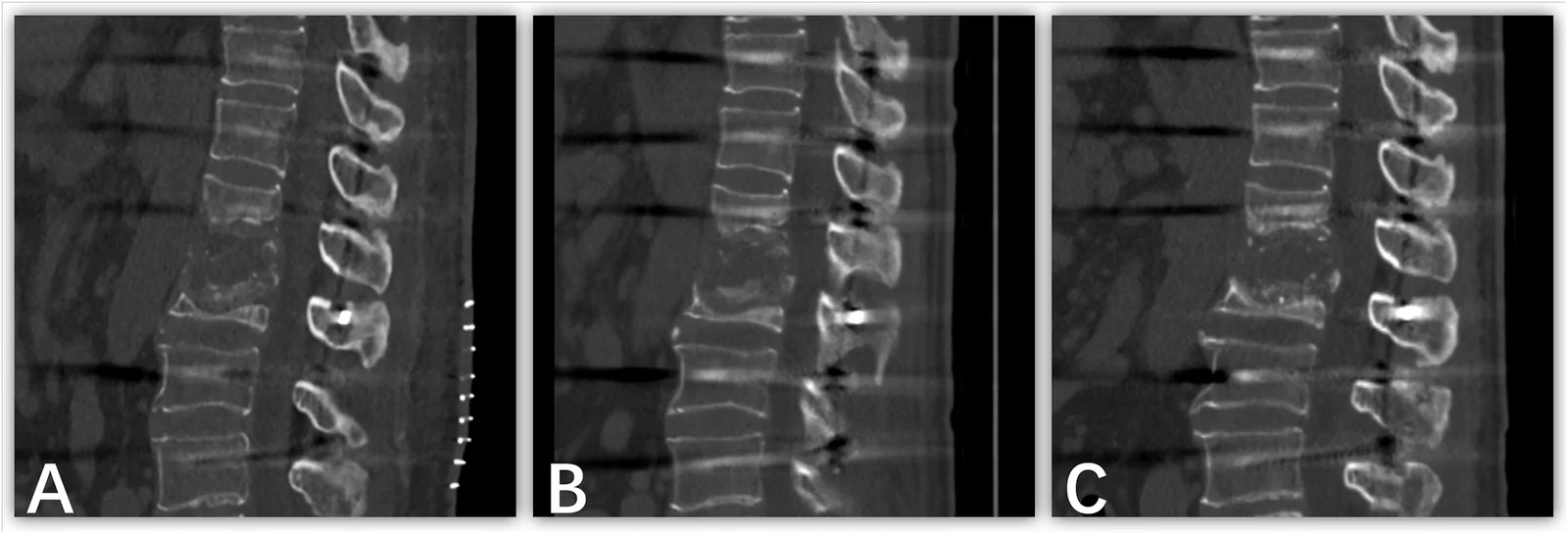
Postoperative CT scans including those at 1 week **(A)**, 1 month **(B)**, and 3 months **(C)** demonstrated that the internal fixation device remains well-positioned, with no evidence of newly developed osteolytic lesions.

## 3 Discussion

WM is a rare B-cell lymphoma characterized by lymphoplasmacytic infiltration in the bone marrow and elevated serum monoclonal IgM (4, 5). The frequency of skeletal involvement in WM depends heavily on the imaging used. In a Danish nationwide population-based cohort of 2,459 patients with WM/LPL, primary bone involvement at diagnosis was recorded in only 1.75% (3). By contrast, a single-centre series from the University of Arkansas for Medical Sciences, in which patients were systematically imaged, reported focal bone disease in approximately 17%–24% on MRI and PET-CT, respectively (6). More recently, osseous involvement at diagnosis was found in 18% of 112 patients with LPL/WM. It included pathological fractures in 4 patients and did not significantly affect response or survival (7). Thus, clinically overt pathological fractures remain uncommon, but subclinical skeletal involvement is probably under-recognized when advanced imaging is not performed routinely. Thus, we provide a concise comparison of the key diagnostic features for differentiating this case from bone metastases of lung cancer, bone metastases of prostate cancer, and multiple myeloma (Table 2, Supplementary Figure S1). In this case, as the patient's pathological fracture involved the intervertebral disc and spinal stability, we chose screw internal fixation instead of percutaneous kyphoplasty surgery.

**Table 2:** Key differential diagnostic criteria between spinal metastases and WM.

Case	Initial symptom	Imaging manifestations	Distinctive Hematological Findings	Treatment strategy
WM	recurrent low back pain of unknown cause	Osteolytic lesion；annular enhancement at the fracture margins	Monoclonal IgM, κ-restricted in ∼75%–80% of cases, λ in the remainder; IgG and IgA often reduced (immunoparesis); IgG/IgA monoclonal bands absent	Stabilization of the fracture site and targeted therapy (BTK inhibitor).
MM (Supplementary Figure S1A)	Widespread ostealgia with generalized skeletal involvement	Diffuse and inhomogeneous bone marrow infiltration.	Serum immunofixation electrophoresis: Monoclonal IgG (∼50%) or IgA (∼20%) paraprotein; light-chain-only myeloma ∼15%–20%; IgD, IgE and IgM rare (IgM <1%). Restricted to κ or λ (κ:λ ≈ 2:1).	Stabilization of the fracture site and chemotherapy.
Bone Metastasis of Prostate Cancer (Supplementary Figure S1B)	Prompt paraplegia of the lower extremities	Extending outward and involving the pedicle, with spinal cord compression.	Prostate specific antigen (PSA) (+), IgG, IgA and IgM usually within the reference range, with normal κ and λ and a normal free κ/λ ratio.	Decompression of the spinal canal, stabilization of the fracture, and palliative management of the primary lesion.
Bone Metastasis OF Lung Cancer (Supplementary Figure S1C)	Low back pain accompanied by lower limb weakness	Diffuse and homogeneous bone marrow infiltration.	Carcino-embryonic antigen (CEA) (+), IgG, IgA and IgM usually within the reference range, with normal κ and λ and a normal free κ/λ ratio	Decompression of the spinal canal, stabilization of the fracture, chemotherapy

The mechanism of bone destruction in WM may be primarily osteolytic rather than due to direct lymphoplasmacytic infiltration, resulting in the disruption of bone microstructure and a decline in vertebral load-bearing capacity. In contrast, both mechanisms contribute to bone destruction in multiple myeloma (8, 9). WM predominantly affects middle-aged and elderly individuals, with a median diagnostic age of approximately 60–70 years, coinciding with the natural decline in bone mineral density. This overlap may exacerbate fracture susceptibility. The patient's age in the present case aligns with this demographic profile, underscoring the need for enhanced differential diagnosis among older adults. Therapeutically, Systemic therapy, including BTK inhibitors and rituximab-based regimens, can effectively reduce tumor burden and alleviate bone pain; however, individualized assessment of comorbidities is essential (10, 11). To identify previously reported cases, we searched PubMed/MEDLINE from inception to 1 July 2026 using the following query: (“Waldenstrom Macroglobulinemia”[MeSH Terms] OR waldenstrom*[Title/Abstract] OR “lymphoplasmacytic lymphoma”[Title/Abstract]) AND (fracture*[Title/Abstract] OR osteolytic[Title/Abstract] OR lytic[Title/Abstract] OR “bone lesion*”[Title/Abstract] OR “bone involvement”[Title/Abstract] OR osseous[Title/Abstract] OR “bone destruction”[Title/Abstract]), restricted to English-language publications. The search yielded 105 records. Titles and abstracts [were screened by two authors (LX and CS), and discrepancies were resolved by a third author (FZ)], followed by full-text review when available. We included case reports of WM or LPL with osteolytic bone lesions and/or pathological fractures that provided individual patient data on the skeletal lesion. We excluded IgM multiple myeloma, coexisting LPL and plasma-cell myeloma, transformation to myeloma, pooled series without individual data, paleopathological studies and non-human reports. Twelve published cases met these criteria; they are summarized together with the present case in Table 3. Two early reports without an abstract (12, 13) were not tabulated. A historical review of 24 cases (14) and a report of mixed lytic and blastic lesions at presentation (15) are cited but not tabulated, because individual treatment data were not available to us. None of the tabulated patients received a BTK inhibitor. BTK inhibition suppresses osteoclast differentiation and function in preclinical models (16), and remineralization of lytic lesions has been reported with ibrutinib in small lymphocytic lymphoma (17). Longer follow-up of our patient is needed to determine whether zanubrutinib confers a similar skeletal benefit in WM.

**Table 3:** Reported cases of WM/LPL with osteolytic lesions or pathological fractures (systematic pubMed search, inception to 1 July 2026).

First author (ref)	Year	Patient	Skeletal presentation	Paraprotein/key findings	Treatment	Outcome (follow-up)
Berman (4)	1975	44 M	Lytic osseous lesions	IgM; predominantly plasmacytic marrow morphology	Radiotherapy; melphalan + prednisone	Some benefit (2 years)
Krausz (21)	1977	NR	Severe osteolytic lesions resembling MM	IgM; associated lymphocytic leukaemia	Chlorambucil	Blood-smear normalization; recalcification of some lesions, but new lytic lesions appeared
Tanaka (22)	1979	M	Lytic lesions of both femora, developing during the course	IgM-κ; preceded by autoimmune haemolytic anaemia	Treatment of AIHA; WM-directed therapy NR	Died of meningeal involvement (autopsy)
Marks (23)	1985	NR	Multifocal skeletal uptake on bone scan	IgM	CVP, later bleomycin + dexamethasone	Unclear (6 months)
Malick (24)	2003	81 F	Lytic lesion of ilium and acetabulum (clinostatic syndrome)	IgM; no high-grade transformation	Radiotherapy	Complete symptom resolution within 1 month
Pujani (9)	2013	65 M	Lytic bone lesions at presentation	IgM-κ; M-spike 5.98 g/dL; CD20 + lymphoid marrow infiltrate	NR	NR
Mehmood (25)	2014	NR	Lytic lesions with hypercalcaemia mimicking MM	IgM; fulfilled WM criteria	Systemic therapy (details NR)	Marked clinical improvement after 2 weeks
Priyanka (26)	2018	NR	Distal tibia and foot	IgM; plasmacytic marrow infiltration	Radiotherapy	Significant symptomatic improvement
Baksh (20)	2021	81 F	Lytic lesions of humeral head, femoral head and acetabulum	IgA-λ (non-IgM) LPL; MYD88 L265P+; biopsy-proven LPL in acetabulum	Rituximab + cyclophosphamide + dexamethasone ×6; rituximab maintenance	Complete response; resolution of lytic lesions on PET-CT
Lin (2)	2022	72 M	L1 compression fracture as first symptom; T-score −2.7	IgM-λ 6.17 g/L; CD5−CD10− clonal B cells; MYD88 L265P+	PVP; declined systemic therapy	NR
Bhatti (3)	2022	65 M	Multiple vertebral fractures (Th7, Th11, Th12, L1, L3) at diagnosis	IgM-κ 6 g/L; MYD88 L265P+	Rituximab + bendamustine ×6; conservative management of spinal fractures	PR with pain relief; stable remission for ∼3 years, then femoral neck fracture and new vertebral fractures → hip prosthesis, radiotherapy (5 Gy × 5) and bisphosphonate; no bone-related symptoms 1 year after surgery
Present case	2026	77 M	Osteolytic fracture of L1–L2 at presentation; osteopenia	IgM-κ 23.10 g/L; free κ/λ 11.659; clustered atypical marrow lymphocytes (24%); CD5−CD10− clonal B cells; MYD88 not tested	Posterior screw fixation; zanubrutinib	No new fracture or osteolytic lesion (3 months)

AIHA, autoimmune haemolytic anaemia; CVP, cyclophosphamide, vincristine and prednisone; F, female; LPL, lymphoplasmacytic lymphoma; M, male; MM, multiple myeloma; NR, not reported; PR, partial response; PVP, percutaneous vertebroplasty.

The case reported by Lin et al. (2) is the closest to ours. A 72-year-old man presented with an L1 compression fracture as the first symptom of WM (IgM-*λ*, 6.17 g/L). Flow cytometry showed CD5(−)CD10(−) clonal B cells, MYD88 L265P was detected, and a vertebral biopsy was obtained during percutaneous vertebroplasty. The diagnosis in that case was better supported by molecular data. Our case, however, differs in several respects. First, Lin's patient had lumbar osteoporosis (T-score −2.7), which may have contributed to the fracture, whereas our patient's bone mineral density was in the osteopenic range. Second, the lesion in our patient involved two contiguous vertebrae (L1–L2) with disc involvement and spinal instability. It therefore required open posterior screw fixation rather than vertebro- or kyphoplasty. This indicates that the surgical strategy should be determined by lesion stability rather than by the underlying haematological diagnosis. Third, the paraprotein burden was substantially higher (IgM-*κ*, 23.10 g/L). Fourth, Lin's patient declined systemic therapy, whereas our patient received first-line zanubrutinib, with no new fracture or osteolytic lesion at 3 months. Finally, the fracture-site biopsy in our case was non-diagnostic, which highlights the need for serum and bone marrow studies and for lesion-directed biopsy when WM is suspected.

Because the fracture-site specimen did not show a clonal infiltrate, we critically considered whether the fracture represents involvement by WM or coincidental coexistence. Several findings favour WM involvement. First, CT showed osteolytic destruction with cortical disruption of two vertebrae. This is not a feature of osteoporotic fractures, and bone mineral density was only osteopenic, with no history of trauma. Second, metastatic carcinoma and myeloma were excluded as far as possible, leaving WM as the only neoplasm identified. Third, WM infiltrates the bone marrow diffusely or interstitially in about 90% of cases (18), and spinal MRI detects vertebral marrow involvement in more than 90% of patients (19). Involvement of the iliac marrow therefore makes involvement of the lumbar vertebral marrow highly probable. Fourth, the fracture-site specimen showed a higher plasma-cell fraction than the iliac aspirate (about 6% vs. 1.0%), with a *κ*-predominant pattern concordant with the serum IgM-*κ* paraprotein. Fifth, when osteolytic lesions in WM/LPL have been biopsied, lymphoma has been found in almost all cases (3, 7, 20). Finally, no new skeletal lesions developed during BTK-inhibitor therapy. Taken together, these findings make a WM-related pathological fracture the most likely explanation. Nevertheless, the fracture-site specimen was non-diagnostic. It was obtained from the fracture line, where necrosis, haemorrhage and repair predominate. Only a plasma-cell/carcinoma panel was applied, so the small B-cell component of WM was not assessed, and decalcification limits light-chain immunohistochemistry. Direct involvement therefore remains probable rather than proven. When WM or another lymphoproliferative disorder is suspected before surgery, we recommend a lesion-directed biopsy away from the fracture line, with B-cell immunohistochemistry (CD20, PAX5, CD79a), light-chain studies and, if possible, MYD88 L265P testing.

## 4 Conclusion

This case shows that an osteolytic lumbar vertebral fracture, most likely caused by osseous involvement, may be the presenting manifestation of WM. Because fracture-site biopsies may be non-diagnostic, a lesion-directed biopsy with B-cell immunohistochemistry and MYD88 L265P testing should be planned when WM is suspected, so that direct skeletal involvement can be confirmed. In clinical practice, WM should be included in the differential diagnosis for patients with vertebral fractures of unknown etiology, accompanied by bone marrow biopsy and serum IgM testing to facilitate early intervention. Further research is warranted to elucidate the molecular mechanisms underlying WM-related bone destruction and explore the potential of targeted therapies.

## Data Availability

The original contributions presented in the study are included in the article/Supplementary Material, further inquiries can be directed to the corresponding authors.

## Supplementary material

The Supplementary Material for this article can be found online at: https://www.frontiersin.org/articles/10.3389/fmed.2026.1947916/full#supplementary-material

Supplementary Figure S1 (A–C): Most characteristic MRI sequences for three spinal metastatic lesions.
